# Temporal Network Pattern Identification by Community Modelling

**DOI:** 10.1038/s41598-019-57123-1

**Published:** 2020-01-14

**Authors:** Xubo Gao, Qiusheng Zheng, Didier A. Vega-Oliveros, Leandro Anghinoni, Liang Zhao

**Affiliations:** 1 Henan Key Laboratory on Public Opinion Intelligent Analysis, School of Computer Science, Zhongyuan University of Technology, ZhengZhou, China; 2 Faculty of Philosophy, Sciences and Letters at Ribeirão Preto (FFCLRP),University of São Paulo (USP), Ribeirão Preto, SP Brazil; 3 Indiana University, School of Informatics, Computing and Engineering, Bloomington, IN USA; 4 Institute of Mathematical and Computer Sciences (ICMC-USP), University of São Paulo (USP), São Carlos, SP Brazil

**Keywords:** Mathematics and computing, Applied mathematics, Computational science

## Abstract

Temporal network mining tasks are usually hard problems. This is because we need to face not only a large amount of data but also its non-stationary nature. In this paper, we propose a method for temporal network pattern representation and pattern change detection following the reductionist approach. The main idea is to model each stable (durable) state of a given temporal network as a community in a sampled static network and the temporal state change is represented by the transition from one community to another. For this purpose, a reduced static single-layer network, called a *target network*, is constructed by sampling and rearranging the original temporal network. Our approach provides a general way not only for temporal networks but also for data stream mining in topological space. Simulation results on artificial and real temporal networks show that the proposed method can group different temporal states into different communities with a very reduced amount of sampled nodes.

## Introduction

In real-world applications, data often arrives in streams and must be analyzed in real-time. Patterns and relations in such data are usually not stable but evolve over time^1,2^. In dynamical and non-stationary environments, the data distribution can change, yielding the phenomenon of concept drift^2,3^. One example is the stock market, where the massive data exchange is strongly related to macro and micro political events, economic events and other factors, such as natural and man-made disasters. Another example is the pattern of customersâ€™ buying preferences, which may depend on the season, availability, inflation rate, etc. Some common types of changes may include a gradual change over time, a recurring or cyclical change, and a sudden or abrupt change. Different concept drift detection and handling schemes may be required for each situation. In this context, uncovering data stream patterns is a fundamental task not only to detect concept drift but also to predict future behaviors. Recently, some works have employed networks as a way for representing data from many real systems^4–7^, leading to complex network research.

Complex network refers to large scale graphs with nontrivial connection patterns^8–11^. One of the most important features of a complex network is the presence of communities. Detecting communities in these systems has become a fundamental task to help us understand how local patterns (represented by sub-networks) interact and produce global behavior. From a network topology point of view, a community is a sub-graph in which the inner links are dense while the outer connections are relatively sparse^4,12,13^. In terms of the information-theoretic field, a community is seen as the module that can diminish or retard the propagation flow of information in the system, for a considerable period of time^14–16^. In the machine learning domain, community detection provides new unsupervised learning methods, where each community corresponds to a data cluster in the clustering problem^4^.

Large systems usually are high-dimensional and heterogeneous in nature, which require a discriminatory, while interactive, structural representation of the organization. In this way, to better improve our understanding of such complex systems, complex network theory has become essential to move beyond simple graphs and to take such multi-type interaction features into account, which include multiple subsystems and layers of connectivity. When a data stream is represented in a network form, it is called as a temporal network^17–21^. A temporal network, also known as a time-varying network, is a network whose links are active only at certain points in time. A great variety of real systems can be represented by networks that evolve over time, examples range from social networks, telecommunication, traffic, climate, and neural-brain networks.

Here, we tackle the concept drift problem proposing a new graph-based method for modeling the system data via temporal networks. Each stable temporal state of the system is represented as a community of the target network, where the concept drift is the community transition. First, our method extracts the target network, which is an intermediate representation of the original network that reduces the dimensionality of the problem by sampling the relevant nodes. In this way, we gain computational performance when calculating several features that are maintained in the target network, like persistence, cyclic pattern, abrupt and gradual changing, etc. Then, the method models each detected concept (or pattern) into a different community. Therefore, we identify not only the change point but also the number of patterns generated by the process.

## Research Problem

We focus on the problem of concept drift in large data-sets, which are represented by temporal networks or data that can be transformed as well. The aim is to propose flexible methods that can adapt to data that evolve or change over time. A similar problem found in the complex network literature is the change point detection^22–25^, which seeks to detect the moments of change between one concept (or network pattern) to the other. In general, this approach focus on measuring the dissimilarity between one snapshot of the temporal network and the next one in time. A change is detected when the dissimilarity passes a fixed threshold^22,23^. These models usually depend on a set of time frames, for which a solution is proposed in^24^. However, in the concept drift scenario, patterns and relations change over time, and fixed models and thresholds may not work after a while. Other methods also rely on probabilistic modeling to detect and predict the changes considering short-time memory^25^. However, the authors did not consider the case of multiple edges placed at any given time, and they focused on inferring a model that reproduces the waiting time in the empirical data.

The method proposed here differs from the previous works mainly in two points: (i) the proposed method models each concept (or pattern) into a different community, which can be accessed after the data is processed (this means that we identify not only the change point but also the number of patterns generated by the process) and (ii) we reduce the dimensionality of the problem by sampling the relevant nodes. We believe that these two characteristics are very relevant for practical purposes since they yield a better understanding of the process (through the number of patterns) with the advantage of not accessing the whole data-set at every timestamp.

When it comes to concept drift, there are two kinds of methods to analyze temporal networks: One is the direct method, where we consider a temporal network as a multiplex network and detect features directly on such ensemble of network’s snapshots. Interesting results have been obtained in this direction. For example, in^19^, the authors presented a set of measures to characterize the dynamical properties of general temporal networks. In^26^, the authors discussed the interpretation of temporal network theory in the context of the dynamic functional brain connectome. Many other studies have been conducted to characterize temporal networks by revealing the community evolution in such networks^5,15,20,27–31^. In multilayer networks, including temporal networks, inter-layer links can potentially change the modular structure of each layer^28^.

In terms of practical use, the contributions of previous works are very relevant in a wide range of real-world applications, like the identification of terrorist groups, tax fraud detection, consumers’ behavior, and the relationship between proteins and genes. In this scenario, the concept (or pattern) in interest can be analyzed through the relationship of the same element among several layers, enabling the community detection in multi-layer networks to potentially provide richer information than the single-layer approach.

To handle a large system, one needs to zoom out from the details. Therefore, the second way to analyze temporal network is to map the original network, usually a large database, to a smaller and easier handled one. Then, we can employ several of the available tools developed so far to analyze the sampled network and draw conclusions to the original large-scale network. In^32^, the authors presented various strategies of temporal network sampling and quantified the biases generated by each sampling strategy on a number of temporal network measures, such as link activity, temporal paths and epidemic spread.

In this work, our method constructs a sampled network, called *target network* from now on, to represent the temporal states of the temporal network. We consider that a temporal network is at a certain state at a time interval. It is expected that the resulting target network contains several communities, each representing a temporal state of the original network and the transition from one community to another yields the concept drift, i.e., the temporal network changes from one state to another. For this purpose, the main steps describing our method are the following:

1. Calculate some network measures for every state node of every physical node;
2. Select a small subset of physical nodes that have the highest levels of fluctuations from the calculated network measures;
3. Run the spatial-temporal model to map the sequence of layers from a temporal network into a reduced (single-layer) target network;
4. Finally, detect the communities from the target network to extract the concepts and concepts drift.

Since the target network just needs to capture the temporal changing pattern of the original network, our sampling method is straightforward and, therefore, the minimization of biases between the sampled network and the original temporal network is no longer the objective^32^.

## Community Detection by Particle Competition

In this section, we briefly introduce the particle competition model, originally proposed in^33^ and improved in^34–36^. When investigated the behavior of this framework, it was showed that a certain level of randomness can largely enhance the learning process, similar to the phenomenon of stochastic resonance, in which the performance of a nonlinear deterministic system can be largely improved by a certain level of noise^4^. Therefore, depending on the system complexity, the use of only deterministic rules might be insufficient to learn the process behind the system^4^. A recent development of this framework showed that it can identify nonlinear features in boundaries between classes with overlapping structural data^36^. Although other community detection algorithms could be selected in this work, the particle competition method was adopted due to its performance in overlapping data and low computational complexity order, which is lower than other network-based semi-supervised algorithms^34,36^. Other than that, this method outputs the participation rate of each node on each community (or how strong a node relates to a certain pattern), enabling us to take that into consideration in further developments of this work.

Let’s consider a network *G* = (*V*,*L*), in which *V* = {*v*_1_, …, *v*_|*V*|_} is the set of nodes and *L* = {*l*_1_, …, *l*_|*L*|_} ⊆ *N* × *N* is the set of links or edges, where *l*_(*i,j*)_ is the weight of the link between nodes *v*_*i*_ and *v*_*j*_ — or {1, 0} for unweighted networks. The particle competition method starts by uniform deploying *K* particles in the network. Each particle is a random walker with the mission of dominating as many nodes as possible while defending its current domain (i.e., nodes) from other particles. Formally, this is a stochastic dynamical process in which the behavioral rules are determined as follow^37^: At each iteration time (*t*), a particle selects a neighbor *v*_*j*_ to visit choosing between a random walking or preferential walking. The latter means, the particle come back to visit a high dominated neighbor. However, the particle lost energy if it visits a node that is in the domain of a rival particle. On the other hand, it re-chargers its energy when visiting an already dominated node by itself. If the particle has energy below a predefined low-energy level, then, the particle becomes exhausted and it will be reset to a randomly selected node in the next iteration. The probabilities of given particle *k* located at node *v*_*i*_ do a random walk (*rand*) or preferential walk (*pref*) are given by Eq. 1 ^37^,

1 $$\begin{array}{rcl}{P}_{rand}^{(k)}({v}_{i},{v}_{j}) & = & \frac{{l}_{(i,j)}}{{\sum }_{{v}_{j}\in V}\,{l}_{(i,j)}},\\ {P}_{pref}^{(k)}({v}_{i},{v}_{j},t) & = & \frac{{l}_{(i,j)}{N}_{j}^{(k)}(t)}{{\sum }_{{v}_{j}\in V}\,{l}_{(i,j)}{N}_{j}^{(k)}(t)}.\end{array}$$

where ${N}_{i}(t)=[{N}_{i}^{1}(t),\,{N}_{i}^{2}(t),\ldots {N}_{i}^{K}(t)]$ is the level of dominance of the particle on each node, in which ${N}_{j}^{(k)}(t)$ is the register of the accumulated number of visits of particle *k* on node ${v}_{j}$ up to iteration $t$. Hence, the transition matrix is defined as the combination of both probabilities, which yield

2 $${P}^{(k)}(t)=\lambda {P}_{pref}^{(k)}(t)+\mathrm{(1}-\lambda ){P}_{rand}^{(k)}(t),$$

where $\lambda \in \mathrm{[0,}\,\mathrm{1]}$ is a balancing parameter. The smaller the $\lambda$, the more randomly the particle walk; The larger the $\lambda$, the more preferential walking it performs. We define the position of the particles at time $t$ as the vector ${\bf{p}}(t)={[{{\bf{p}}}^{(1)}(t),{{\bf{p}}}^{(2)}(t),\ldots {{\bf{p}}}^{(K)}(t)]}^{T}$ and their energy as the vector $E(t)={[{E}^{(1)}(t),{E}^{(2)}(t),\ldots {E}^{(K)}(t)]}^{T}$. In this way, the particle competition dynamical system is described by the following equations:

3 $${{\bf{p}}}^{(k)}(t+\mathrm{1)}={v}_{j},\,{v}_{j} \sim {P}^{(k)}(t),$$

4 $${N}_{i}^{(k)}(t+\mathrm{1)}={N}_{i}^{(k)}(t)+{1}_{{p}^{(k)}(t+\mathrm{1)}=i},$$

5 $${E}^{(k)}(t+\mathrm{1)}=\{\begin{array}{lll}min({\omega }_{max},\,{E}^{(k)}(t)+\Delta ) & {\rm{if}} & {\rm{owner}}\,(k,t)\\ max({\omega }_{min},\,{E}^{(k)}(t)-\Delta ) & {\rm{if}} & \neg {\rm{owner}}\,(k,t)),\end{array}$$

6 $${S}^{(k)}(t+\mathrm{1)}=\delta ({E}^{(k)}(t+\mathrm{1),}\,{\omega }_{min}),$$

where $S(t)={[{S}^{(1)}(t),{S}^{(2)}(t),\ldots {S}^{(K)}(t)]}^{T}$ is the $\delta$ function that indicates whether or not the particle is active or exhausted.

For each of the $K$ particles, a neighbor node is selected using the transition matrix by Eq. 2. Then, the target node of each particle is determined by Eq. 3 and the number of visits (domination level) by Eq. 4. After that, the energy levels of particles is updated by Eq. 5. Finally, we check whether each particle is active or exhausted by Eq. 6. If the particle is with enough energy, it continuous walking in the network; otherwise, the particle is reset to a randomly selected node and its energy level is recharged to the maximal level. This process is repeated until the system converges, when it is expected that each particle dominates a set of nodes corresponding to a community of the network.

## Method

In this section, we present the method of the static reduced network (target network) construction from a usually very large temporal network. After that, the community detection is performed on the target network and a method for finding out the best number of communities is also proposed.

### Target network construction

Given a temporal network, *G*(*t*) = (*V*, *L*(*t*)), we firstly transform it into a reduced single-layer network (target network), *G*_*r*_ = (*V*_*r*_, *L*_*r*_), preserving the spatial-temporal pattern of *G*(*t*). In a temporal network, there are *N* physical nodes *v*_*i*_, *i* = 1, 2, …, *N* and each of them has *T* state nodes *v*_*i*_(*t*), *t* = 1, 2, …, *T*. For each state network of the original temporal network, we calculate network measures for each state node. Many metrics can be used, such as communicability, betweenness, Katz centrality and PageRank^4,38^. In our method, we use the communicability measure as described in^38^, which accounts not only for the shortest paths connecting two nodes but also the longer paths with a lower contribution. In spite of the high computational complexity of this measure in comparison to some other alternatives, like the PageRank, we understand that the sampling step of our method is not critical for its overall applicability since the sampling is done once for each segment of data that contains several time instants. Therefore, we chose the metric that presented better overall results. The communicability ${M}_{{v}_{i}}$ from node ${v}_{i}$ to all other nodes of the network is described by,

7 $${M}_{{v}_{i}}=\frac{1}{(N-\mathrm{1)}}\sum _{j\in N}\,(\frac{1}{{\ell }_{i,j}!}\,\Phi \,({v}_{i},{v}_{j},\,{\rm{\min }})+\sum _{z > {\ell }_{i,j}}\,\frac{1}{k!}\Phi ({v}_{i},{v}_{j},z)),{v}_{i}\ne {v}_{j}$$

where for nodes ${v}_{i}$ and ${v}_{j}$, ${\ell }_{i,j}$ is the shortest path length between the nodes, $\Phi ({v}_{i},{v}_{j},min)$ is the number of shortest paths and $\Phi ({v}_{i},{v}_{j},z)$ is the number of paths connecting ${v}_{i}$ and ${v}_{j}$ of size $z > \ell$. The reasoning behind this choice is that the shortest paths are significantly affected by structural changes in a network.

At this step, each state network is treated separately and the evolution nature is ignored, therefore ${M}_{{v}_{i}}$ is calculated for each node of each state network. With these values at hand, we start to construct the target network. For this purpose, we divide the modeling into three phases:

1. Sampling. For each physical node ${v}_{i}$, we calculate the standard deviation of the state network measure. Considering the communicability measure, ${\sigma }_{{v}_{i}}$ is the standard deviation of the values ${M}_{{v}_{i}(t)}$, $T=1,2,...,T$. Then, we select the $P$ physical nodes with the highest standard deviation to create the target network. $P$ is empirically defined. Our simulation results show that usually *P* ≪ *N*, which means that the original network size can be largely reduced and the target network still captures its dynamics. The resulting target network will have the following elements:
  8 $${v}_{r}=[\begin{array}{cccc}{v}_{{r}_{1}}\mathrm{(1)} & {v}_{{r}_{1}}\mathrm{(2)} & \mathrm{..}. & {v}_{{r}_{1}}(T)\\ {v}_{{r}_{2}}\mathrm{(1)} & {v}_{{r}_{2}}\mathrm{(2)} & \mathrm{..}. & {v}_{{r}_{2}}(T)\\ \mathrm{..}. & \mathrm{..}. & \mathrm{..}. & \mathrm{..}.\\ {v}_{{r}_{P}}\mathrm{(1)} & {v}_{{r}_{P}}\mathrm{(2)} & \mathrm{..}. & {v}_{{r}_{P}}(T)\end{array}]\mathrm{}.$$
  where $P < N$ (usually $P <  < N$) is the number of selected physical nodes. At this stage we want to find the temporally stable pattern of the input network, therefore, we just make spatial sampling, while maintain the whole temporal sequence of selected nodes. The sampling process is illustrated in Fig. 1.

**Figure 1:**
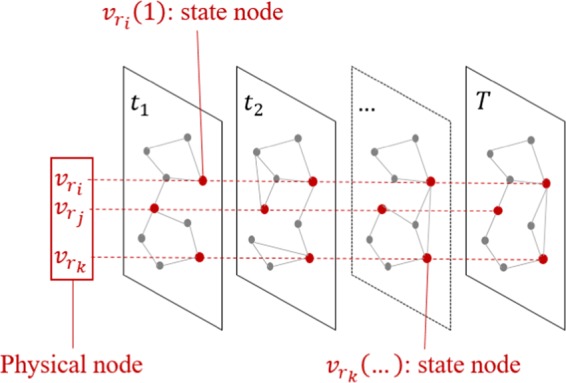
Sampling process illustration. For each physical node, the predefined network measure and its standard deviation along time are calculated and only the $P$ nodes with the highest standard deviation values are selected. In this figure a small example is depicted, where three nodes are selected (in red). ${v}_{{r}_{i}}$, ${v}_{{r}_{j}}$ and ${v}_{{r}_{k}}$.
2. Temporal coding. For each selected physical node ${v}_{{r}_{i}}$, we calculate the similarity of network measures of every pair of the state nodes, ${S}_{i}({t}_{l},{t}_{m}),i,m=1,2,...,T,l\ne m$. Then, each pair of state nodes, say nodes ${v}_{{r}_{i}}({t}_{l})$ and ${v}_{{r}_{i}}({t}_{m})$, receives a weight ${C}_{1}{S}_{i}({t}_{l},{t}_{m})$, where ${C}_{1}\in \mathrm{[0,}\,\mathrm{1]}$ is a parameter to define the influence strength of temporal feature in the final target network. In this way, we construct $P$ separated networks. Figure 2 illustrates the relationship of the introduced symbols regarding the selected physical node ${v}_{{r}_{i}}$ and its states.

**Figure 2:**
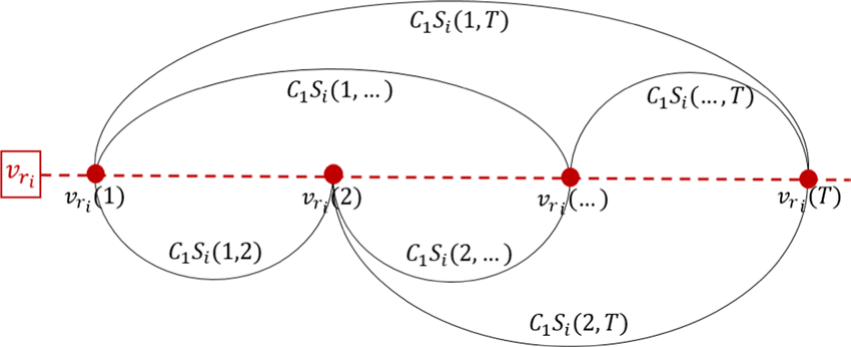
Temporal coding illustration. In this example, we show the network constructed from temporal relations of node ${v}_{{r}_{i}}$. The weights are the similarities between the communicability measure at each time instant. At this stage, the state nodes of each selected physical node from the sampling process form an isolated network. Therefore, $P$ networks are constructed at this stage and these will be connected in the spatial coding process.
3. Spatial coding. In a similar way, the similarity of network measures between every pair of spatially selected nodes, say nodes ${v}_{{r}_{i}}({t}_{l})$ and ${v}_{{r}_{j}}({t}_{m})$, is calculated, ${S}_{ij}({t}_{l},{t}_{m}),i\ne j$. Correspondingly, the weight of connection between ${v}_{{r}_{i}}({t}_{l})$ and ${v}_{{r}_{j}}({t}_{m})$ is ${C}_{2}{S}_{ij}({t}_{l},{t}_{m})$, where the constant ${C}_{2}$ characterizes the influence strength of spatial correlation in the target network. Figure 3 illustrates the introduced symbols in the sampled network at the spatial coding stage.

**Figure 3:**
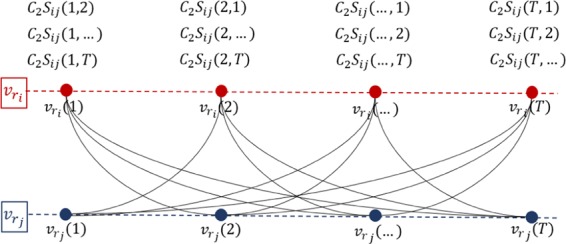
Spatial coding illustration. In this example we show the connections generated from node ${v}_{{r}_{i}}$ to ${v}_{{r}_{j}}$. This stage reconnects the $P$ networks constructed during the temporal coding stage generating the final target network.

According to the network construction criteria, we expect that the network presents community structure, where each community represents a system state and the concept drift happens when the process changes from one community to another. For this purpose, we detect communities of the target network ${G}_{r}=\langle {V}_{r},{E}_{r}\rangle$. Specifically, the particle competition method is used here to find out communities due to its robustness, efficiency and rich information generated during the detection process.

### Discovering the best number of communities

To discover the best division of the network into communities using the particle competition method, it is necessary to determine the optimal number of particles $K$. For this purpose, we describe the localized average domination level measure, previously introduced by this author^37^:

9 $$\begin{array}{rcl}\langle R(K)\rangle  & = & min\{\frac{1}{{V}_{1}}\sum _{u\in {N}_{1}}{({N}_{u}^{1}(\infty ))}_{max},\ldots ,\\  &  & \frac{1}{{V}_{k}}\sum _{(u\in {N}_{k})}{({N}_{u}^{k}(\infty ))}_{max},\ldots ,\\  &  & \frac{1}{{V}_{K}}\sum _{(u\in {N}_{K})}{({N}_{u}^{K}(\infty ))}_{max}\,\},\end{array}$$

where ${({N}_{{u}^{k}}(\infty ))}_{max}$ indicates the domination level of particle $k$ on node $u$ at the end of the particle competition process. The subscript $max$ means that particle $k$ has the highest domination level on $u$ among all particles. ${N}_{k}$ is a set of nodes, in which particle $k$ has dominance.

In terms of process, first we calculate the average highest domination level for the nodes occupied by each particle. Then we choose the minimum of the $K$ average highest domination levels as the proposed measure $\langle R(K)\rangle$. The reasoning behind this strategy can be intuitively inferred by the following examples. If we put exactly $K$ particles in a network with $K$ communities, each particle is expected to dominate a community. In this case, the maximal domination level of the nodes $\langle R(K)\rangle$ will be high. If, however, we put more particles than the number of communities, at least one community will have more than one particle competing for dominance, resulting in a low value of $\langle R(K)\rangle$. On the other hand, if we put less than $K$ particles in the network, the competition within the same community also happens, due to the equal behavior of all particles. So, again, $\langle R(K)\rangle$ will be a low value.

Therefore, the optimal number of particles $K$ will result in the highest value of $\langle R(K)\rangle$. In practical terms, we check the value of $\langle R(K)\rangle$ by putting $2$ to an estimated ${K}_{max}$ number of particles to the network and running the particle competition method until it converges. After that, we calculate the measure $\langle R(K)\rangle$. The best number of particles ${K}_{best}$ is the one where $\langle R(K)\rangle$ reaches the maximal value:

10 $${k}_{best}=\,{\max }\,(\langle R(1)\rangle ),(\langle R(2)\rangle ),\ldots ,(\langle R({k}_{{\max }})\rangle ).$$

## Experimental Results

In this section, we present the simulation results applying the proposed method for temporal network pattern characterization. Three experiments were performed in synthetic temporal networks. In the first experiment, the input temporal network presents one structural change; in the second experiment, three structural changes with repetition; and in the third experiment, gradual changes. These scenarios simulate abrupt, cyclical and gradual pattern changes in temporal networks.

In all the cases, each state network is generated by the following rule: a pair of nodes is connected with probability ${p}_{in}$ if they are in the same community, whereas a pair of nodes belonging to different groups are connected with probability ${p}_{out}$. We choose ${p}_{in}$ and ${p}_{out}$ in order to control the number of intracommunity links ${z}_{in}$ and the number of inter-community links ${z}_{out}$ for a given network average degree $\langle k\rangle$. Based on these parameters, we can define the fraction of intra-community links ${z}_{in}/\langle k\rangle$ and the fraction of inter-community links ${z}_{out}/\langle k\rangle$ of the network, where ${z}_{in}/\langle k\rangle +{z}_{out}/\langle k\rangle \mathrm{=1}$.

**Figure 4:**
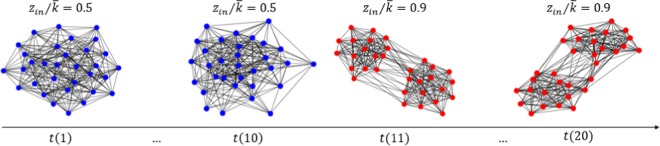
Input temporal network used in the first experiment. Generated in Python (Python Software Foundation. Python Language Reference. Available at http://www.python.org).

In the first experiment, the input temporal network consists of $20$ random cluster networks each with $32$ nodes divided into $2$ communities. The first $10$ state networks are generated with a value of inter-cluster parameter ${z}_{in}$, while the other $10$ state networks are generated with a different ${z}_{in}$ value. It means that the first $10$ networks represent a temporal state, while the second $10$ represent another state. This input network is illustrated in Fig. 4.

To generate the target network, we calculate the communicability measure for each state node and only $5$ nodes of each network with the largest temporal standard deviation of communicability values are chosen. Therefore, each target network contains 5 × 20 = 100 nodes. According to our method, those $100$ nodes should be divided into $2$ communities each representing a temporal state of the original network.

**Figure 5:**
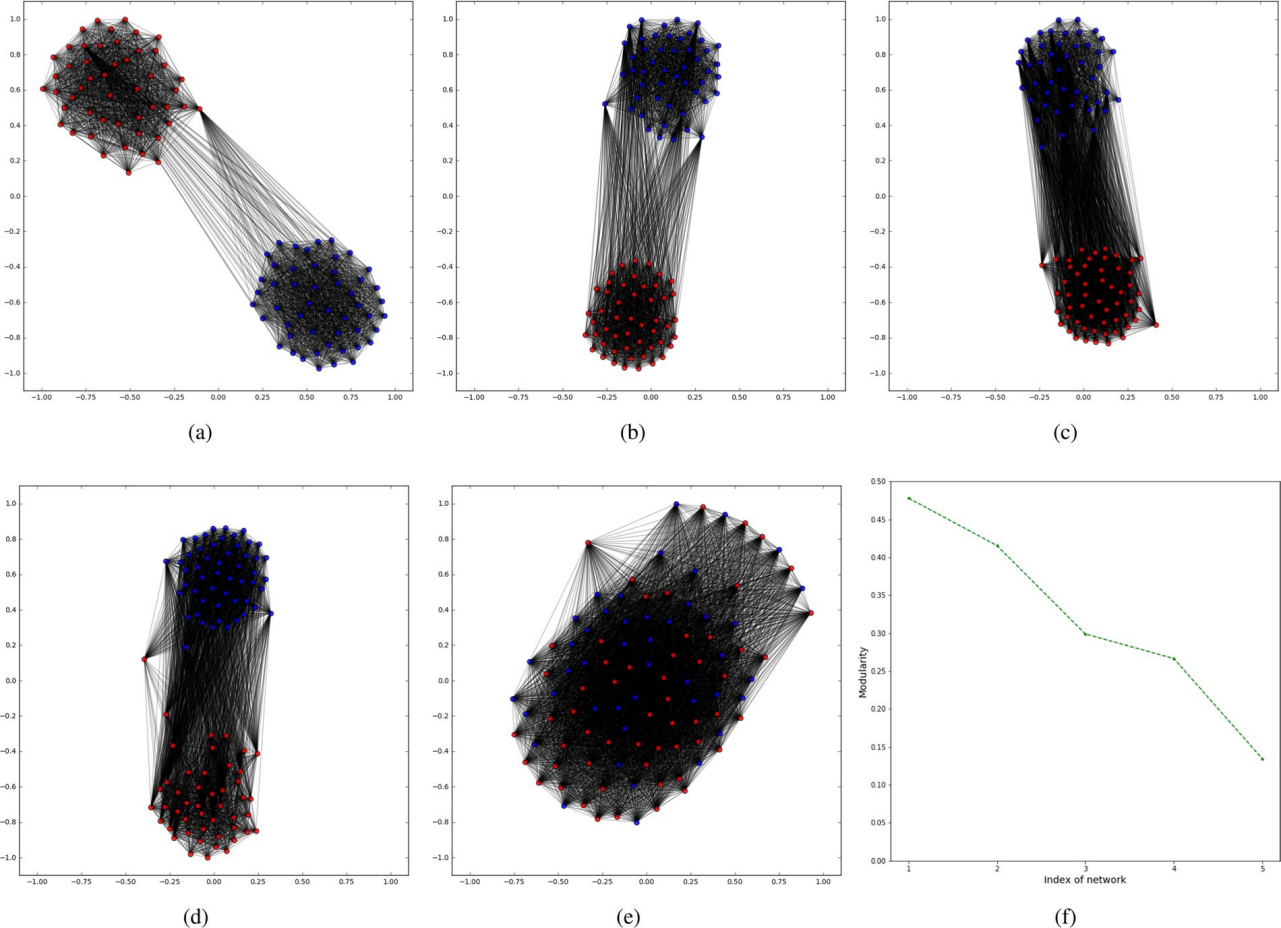
Target networks and their respective detected communities. In these simulations, each state network is a random clustered network and it is generated using the parameter corresponding to: (**a**) for the first 10 state networks, *z*_*in*_/〈*k*〉 = 0.5. For the second 10 state networks, *z*_*in*_/〈*k*〉 = 0.9; (**b**) for the first 10 state networks, *z*_*in*_/〈*k*〉 = 0.55. For the second 10 state networks, *z*_*in*_/〈*k*〉 = 0.85; (**c**) for the first 10 state networks, *z*_*in*_/〈*k*〉 = 0.6. For the second 10 state networks, *z*_*in*_/〈*k*〉 = 0.8; (**d**) for the first 10 state networks, *z*_*in*_/〈*k*〉 = 0.65. For the second 10 state networks, *z*_*in*_/〈*k*〉 = 0.75; (**e**) for the first 10 state networks, *z*_*in*_ = 0.7. For the second 10 state networks, *z*_*in*_ = 0.7; (**f**) the Modularity calculated for the reduced networks from (**a**–**e**). Generated in Python (Python Software Foundation. Python Language Reference. Available at http://www.python.org).

Five different scenarios are tested with different values of ${z}_{in}$. This example (Fig. 5) shows the abrupt changing situation. In these simulations, each state network is a random cluster network with $N$ nodes equally divided into $M$ groups^39^.

From Fig. 5(a), we see clearly the two communities each representing a temporal state. From Fig. 5(a–d), the two states of the temporal network are closer, therefore, the inter-connection of the two communities are denser. Specially, Fig. 5(e) doesn’t present any community structure because the first $10$ and the second $10$ networks have the same structure, with ${z}_{in}/\langle k\rangle =0.7$.

As we expected, Fig. 5(f) shows that the modularity values, which correspond to the target networks of Fig. 5(a–e), decrease as the two temporal states of each case become closer.

**Figure 6:**
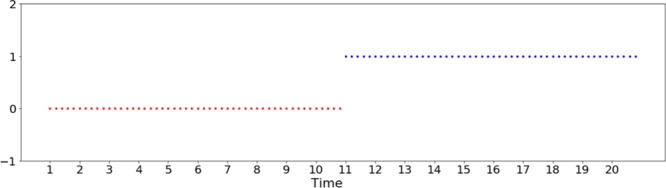
The temporal state evolution of all selected nodes of the first experiment (abrupt change simulation). Each line represents a community.

Figure 6 shows the pattern (community) evolution of the first temporal network. Here, all the selected nodes over time are plotted. The value of each node represents the community number, to which it belongs. We see that the first $10$ networks form a state, while the second $10$ networks form another state.

**Figure 7:**
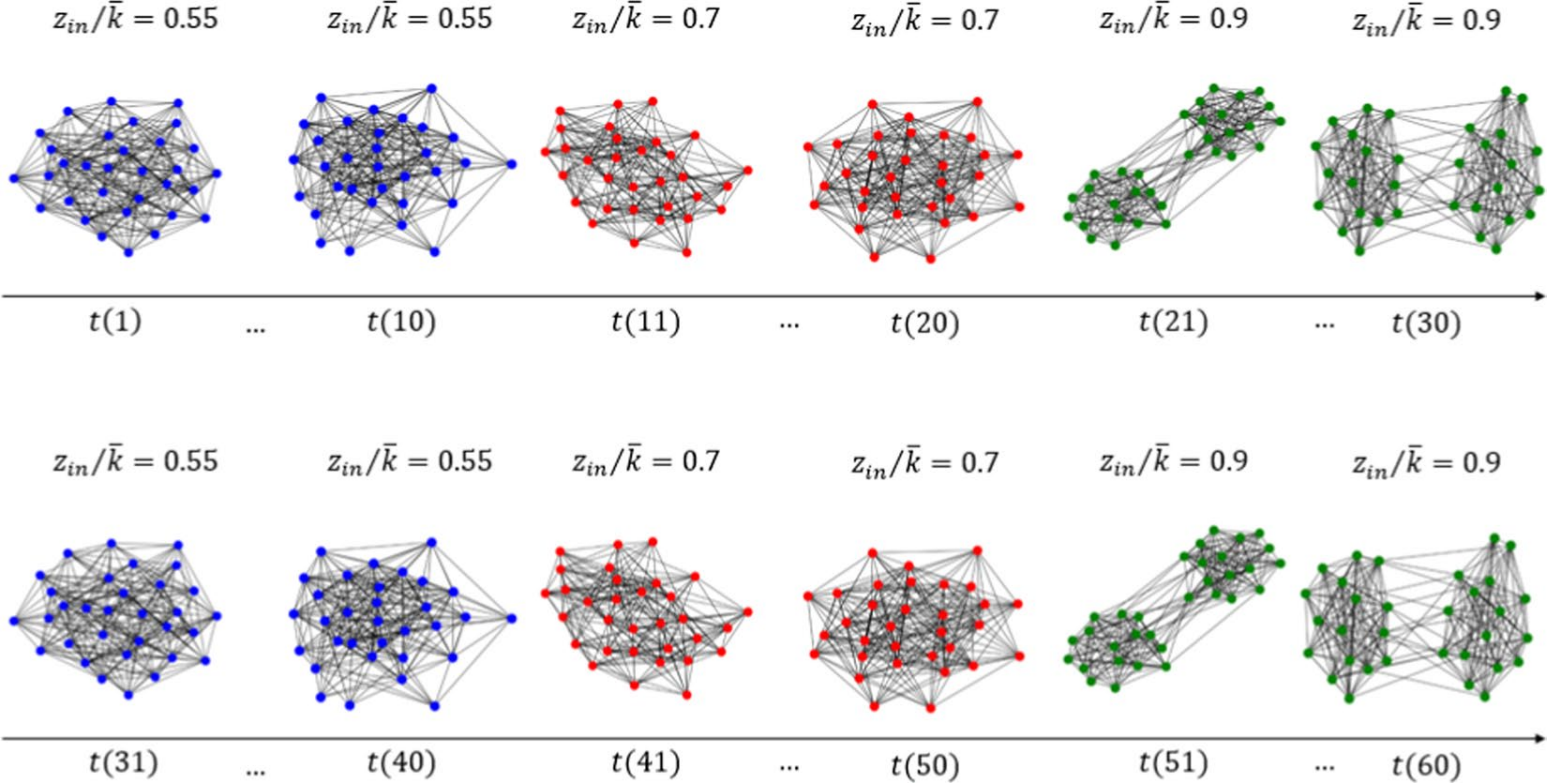
Input temporal network used in the second experiment. Figures generated in Python (Python Software Foundation. Python Language Reference. Available at http://www.python.org).

**Figure 8:**
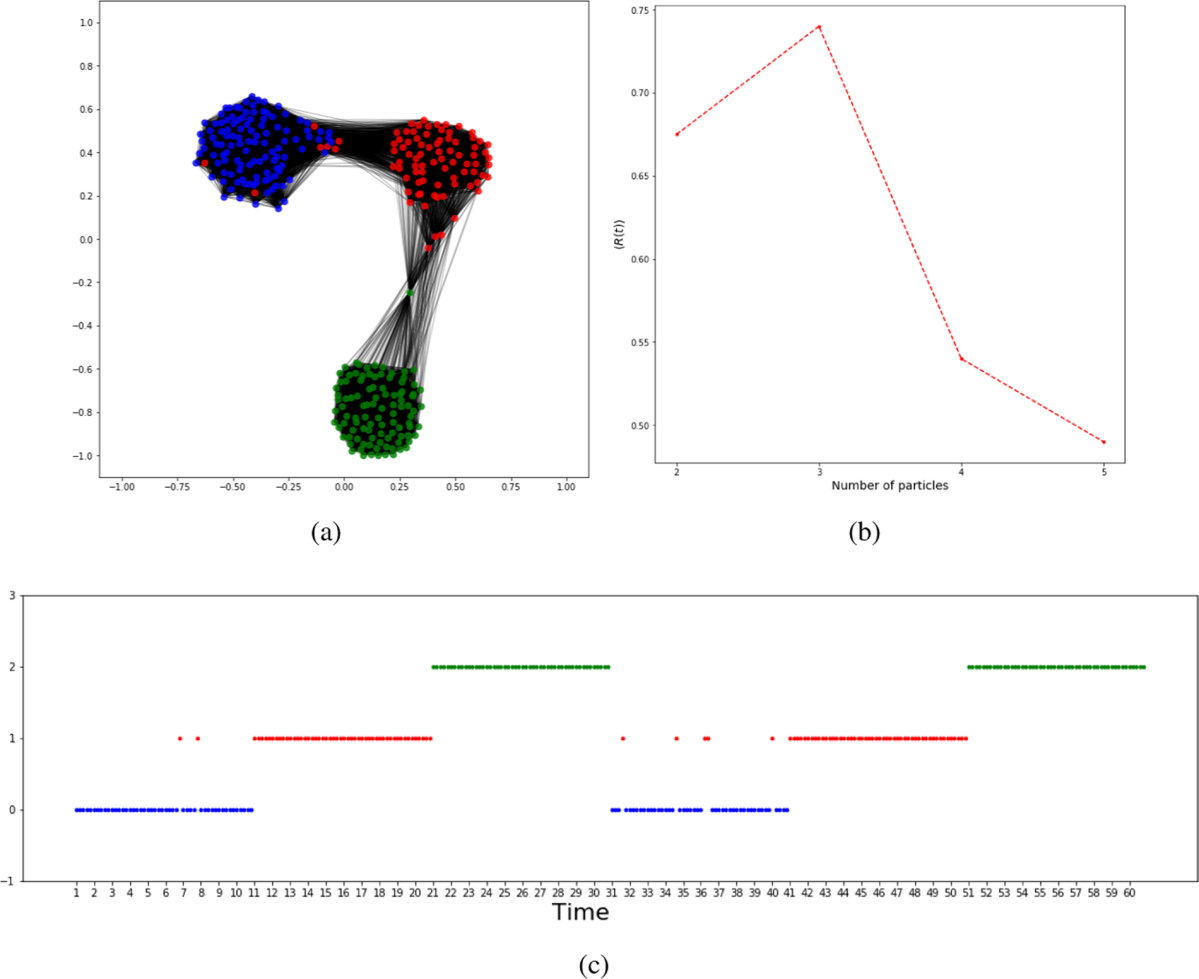
The target network and the communities detected: (**a**) for the first 10 state networks, *z*_*in*_/〈*k*〉 = 0.55; for the second 10 state networks, *z*_*in*_/〈*k*〉 = 0.7, and for the third 10 state networks, *z*_*in*_/〈*k*〉 = 0.9. Then this pattern is repeated once; (**b**) the localized average domination level in relation to the number of particles used in the particle competition process; (**c**) the temporal state evolution of all selected nodes in this experiment (cyclic pattern change simulation). Each line represents a community. Figures generated in Python (Python Software Foundation. Python Language Reference. Available at http://www.python.org).

The second example intends to show how the proposed method represents and detects periodic patterns of a temporal network. The input network consists of $10$ random cluster networks with ${z}_{in}/\langle k\rangle =0.55$, $10$ random cluster networks with ${z}_{in}/\langle k\rangle \,=\,0.7$, $10$ random cluster networks with ${z}_{in}/\langle k\rangle =0.9$, and this pattern repeated once (Fig. 7). In total, we have $60$ networks in $3$ states. Again, only $5$ nodes are selected for each state network. Figure 8(a) shows the target network and we see the three correctly detected communities.

Figure 8(b) shows that the localized average domination level reaches its maximal value when $3$ particles are put in the network in the community detection process, indicating that the three community structure is the best way to partition the target network.

Figure 8(c) shows the temporal evolution of all selected nodes regarding their respective communities. We see three different states (communities) and such states repeated once. The results match well the temporal network design.

**Figure 9:**
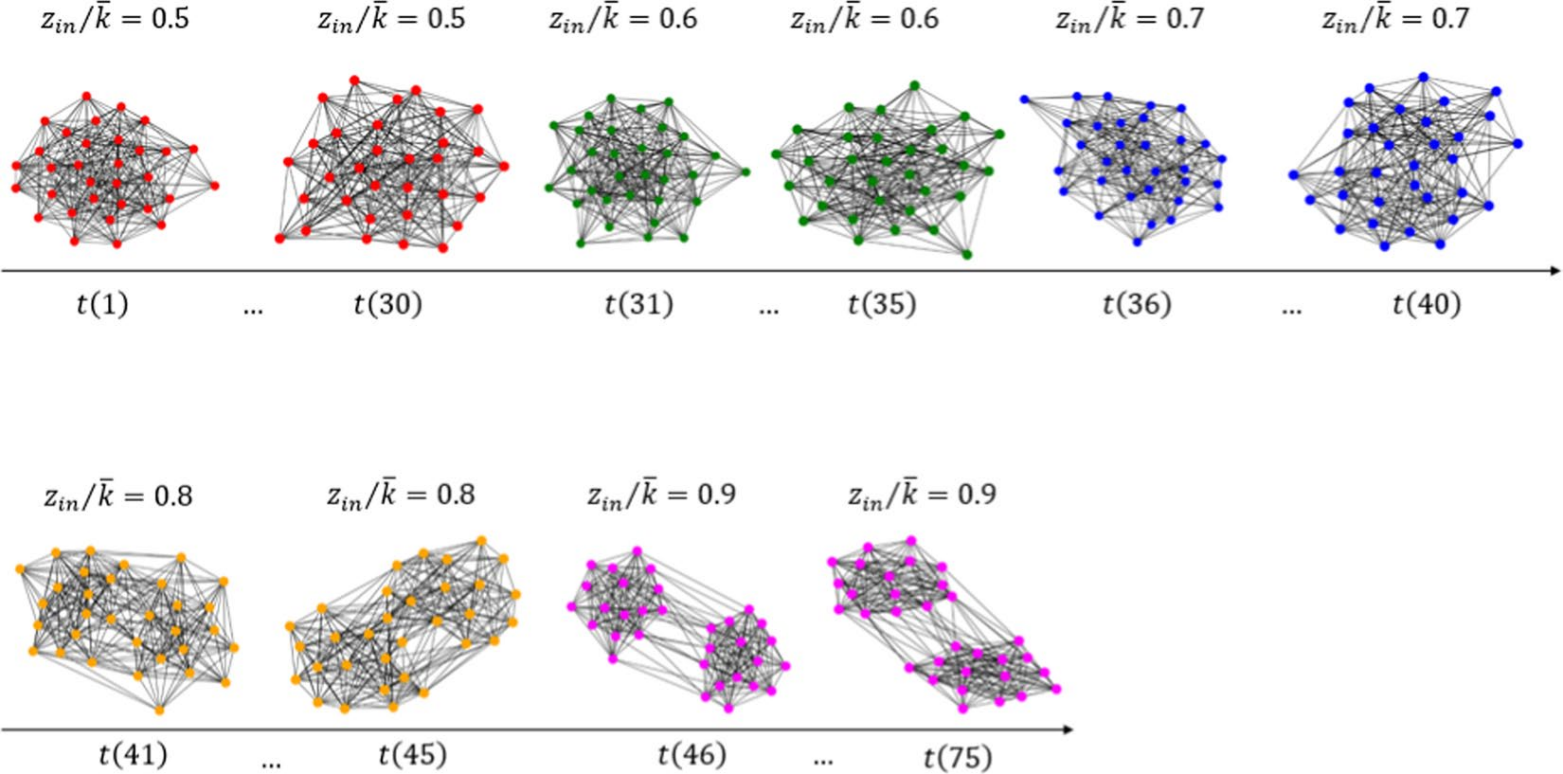
Input temporal network used in the third experiment. Generated in Python (Python Software Foundation. Python Language Reference. Available at http://www.python.org).

**Figure 10:**
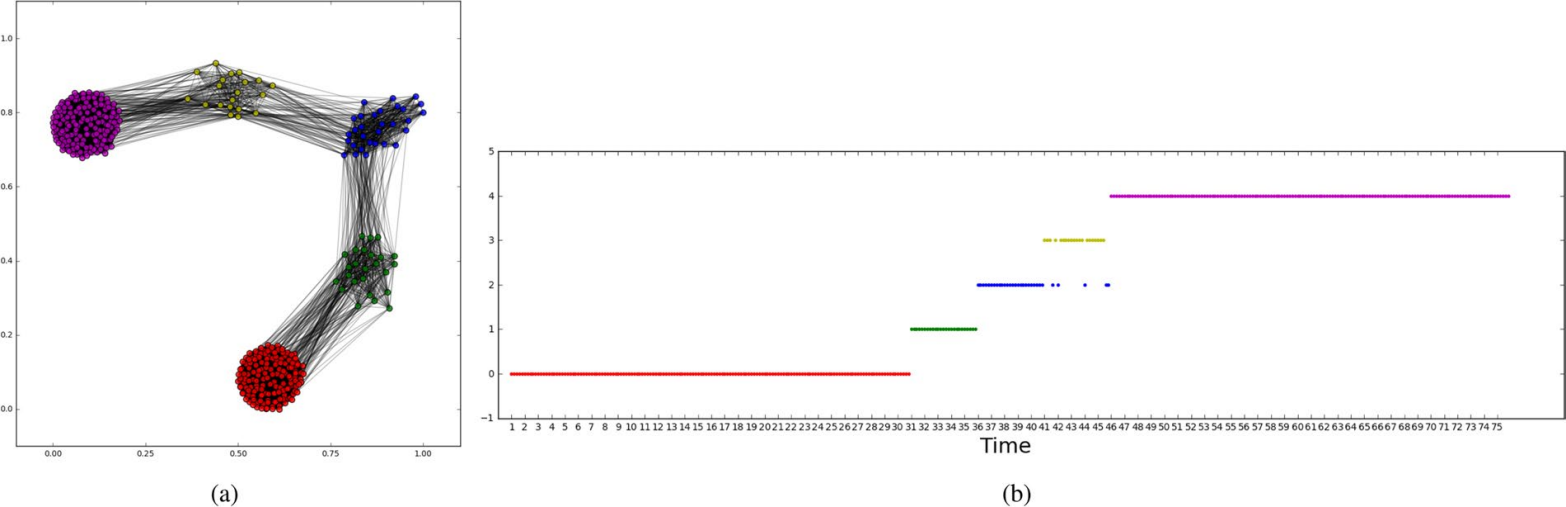
The target network and the communities detected for the gradually changed temporal network (the third experiment). (**a**) For the first 30 state networks, *z*_*in*_/〈*k*〉 = 0.5; for the second 5 state networks, *z*_*in*_/〈*k*〉 = 0.7; for the third 5 state networks, *z*_*in*_/〈*k*〉 = 0.7; for the fourth 5 state networks, *z*_*in*_/〈*k*〉 = 0.8; and for the fifth 30 state networks, *z*_*in*_/〈*k*〉 = 0.9. (**b**) The temporal state evolution of all selected nodes of this experiment. Each line represents a community. Figures generated in Python (Python Software Foundation. Python Language Reference. Available at http://www.python.org).

The third example presents the community structure of the gradually changed temporal network. The input network consists of 30 random cluster networks with *z*_*in*_ = 0.5, 5 random cluster networks with *z*_*in*_ = 0.6, 5 random cluster networks with *z*_*in*_ = 0.7, 5 random cluster networks with *z*_*in*_ = 0.8, and 30 random cluster networks with *z*_*in*_ = 0.9. In total, we have 75 networks (Fig. 9). The first and the last groups of networks represent two durable states, while the three middle groups represent gradual changing states. Again, only 5 nodes are selected for each state network. Figure 10(a) shows the target network and we see the 5 correctly detected communities. The two big communities correspond to the two durable states and the three small communities correspond to intermediate states. It means that a temporal network undergoes an abrupt change if the state transition occurs directly between big communities, while a gradual change happens if the state transition occurs from one big community to another through various small communities. Figure 10(b) shows the community evolution of the target network with long-term states and three intermediate short-term states.

**Figure 11:**
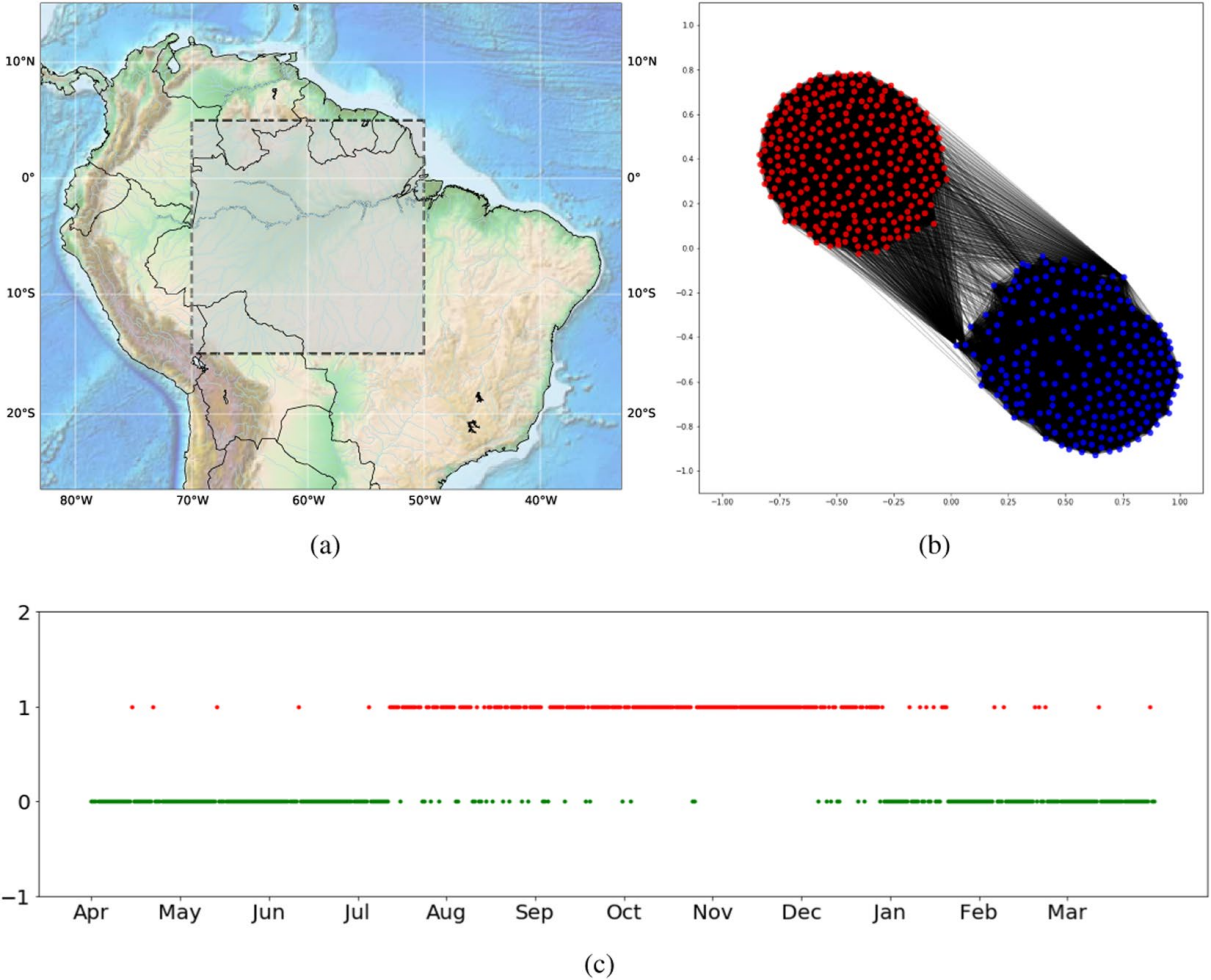
Real fire events data of the Amazon-basin, from first May 2011 to April 31, 2012. (**a**) The selected area (inner rectangle) is delimited from latitude ${5}^{\circ }$N to ${15}^{\circ }$S and from longitude ${70}^{\circ }$W to ${50}^{\circ }$W. (**b**) The target network and the two detected community of the wildfire temporal network. (**c**) The temporal state patterns of the wildfire events according to temporal network. Figure (**a**) was generated using Basemap Matplotlib (https://matplotlib.org/basemap/)^41^; figures (**b,c**) were generated using Python (Python Software Foundation. Available at http://www.python.org).

We test our method with a public available real-world temporal network dataset related to fires events^5^. In a global scale, the fire event activity is collected mainly through satellite instruments like the Moderate Resolution Imaging Spectroradiometer (MODIS), and the dataset is specifically from the Global Daily Fire Location Product (MCD14ML)^40^. The MODIS runs in both, Aqua and Terra satellites, which are operated by the National Aeronautics and Space Administration (NASA). In this research, we use the last version (C6) of MODIS. The studied period is between 01 May 2011 and 31 April 2012 and the region under study is a portion of the Amazon basin, that is located between longitude ${70}^{o}W$, ${50}^{o}W$ and latitude ${15}^{o}S$, ${5}^{o}N$ (Fig. 11(a)). This region is divided into $900$ grid cells, in which each one is a physical node in the constructed network.

The network characterization process for spatio-temporal events is based on these three steps^5^:

- Grid-division: A geographical region under consideration is divided into a grid. Each grid cell is represented as a node in the network. We divided the studied area in a grid of 30 × 30 cells.
- Time interval: The network data modeling can be defined for specific periods. Specifically, the networks were constructed in consecutive intervals of seven days.
- Links: From the data set, two successive fire events create a link between the grid cells where they are located.

Since the Amazon basin is mostly located at the south-hemisphere, the period from July to December corresponds to the dry season with a high tendency of fires and the period from January to June corresponds to the wet season, with a low frequency of fires^5,42^. Figure 11(b) shows the two communities detected in the target network, one represents the season with high wildfire activity and the other represents the one with the low wildfire activity. In this simulation, just $10$ among $900$ physical nodes were selected to construct the target network. Figure 11(c) shows the node-community disposition over time, where the low and high wildfire states are identified by our method. Note that the points outside of the temporal state patterns are explained by the fraction of cells where happen wild-fires and are located in the north-hemisphere, i.e., in a different temporal phase^5,42^.

## Discussion

In this work, we have presented a reductionist method to treat temporal networks, usually composed of large data-sets. Moreover, each durable temporal state is modeled as a community of the target network and the concept drift is represented by the transition from one community to another. There are two approaches to treat temporal networks. On one hand, one can handle the temporal changing problem directly to detect communities of the temporal network and analyze the community evolution. However, this approach presents high complexity and high computational cost. On the other hand, one can just extract some statistical measures from the temporal network to characterize the temporal changing. But, such characterization simplifies too much of the original network and we may need a set of measures to represent the states. Our method brings a trade-off of both approaches, in which an intermediate representation (the sampled target network) is generated. In this way, our method is computationally efficient while many features of the original temporal network, like persistence, cyclic pattern, abrupt and gradual changing, are still maintained in the target network.

One important product of this work is that the communities detected in the target network represent the patterns contained in the data segment. This means that not only can we identify the changes in the data concept, but we can study the patterns and their relations in the constructed target network. In future works, this property can be used to evaluate other behaviors not explored here, like pattern recurrence, pattern stability, and even some predictive model based on node location, diffusion and link prediction^43^.

Finally, this framework can be adapted while maintaining the general idea. Although the use of the particle competition method yielded good results, in principle, any community detection method could be used in its place, e.g., Louvain^44^ or InfoMap^16^. Thus, one could, for example, use a different similarity measure or a different community detection technique depending on the problem domain. Moreover, it can be applied to other real data-sets, including different types of data, such as multivariate time series.
